# Angiotensin-Converting Enzyme Inhibitors or Angiotensin Receptor Blockers After Transcatheter Aortic Valve Replacement: A Meta-Analysis

**DOI:** 10.1016/j.jacadv.2024.100927

**Published:** 2024-04-06

**Authors:** Vivek Bhat, Ashish Kumar, Ankur Kalra

**Affiliations:** a Department of Internal Medicine, St. John’s Medical College, Bangalore, India; b Department of Internal Medicine, Cleveland Clinic Akron General, Akron, Ohio, USA; c Franciscan Health, Lafayette, Indiana, USA; d Krannert Cardiovascular Research Center, Indiana University School of Medicine, Indianapolis, Indiana, USA

**Keywords:** aortic stenosis, renin-angiotensin-aldosterone system, structural heart disease, valve implantation

## Abstract

**Background:**

Persistent left ventricular hypertrophy after transcatheter aortic valve replacement (TAVR) has been associated with poor outcomes. Angiotensin-converting enzyme inhibitors (ACEIs) and angiotensin receptor blockers (ARBs), due to their favorable effects on ventricular remodeling, have been hypothesized to improve outcomes post-TAVR, yet there are no recommendations regarding their use.

**Objectives:**

This study aimed to compare the outcomes of patients receiving ACEIs/ARBs with those not receiving ACEIs/ARBs after TAVR.

**Methods:**

We performed a literature search on PubMed and Cochrane Library until June 14, 2023, and included all studies comparing clinical outcomes between patients given ACEIs/ARBs and those not given ACEIs/ARBs after TAVR. All-cause mortality was the primary outcome. We used a random effects model with appropriate corrections to calculate relative risk (RR) and CIs, with all analyses carried out using R v4.0.3.

**Results:**

We included ten studies on the use of ACEIs/ARBs post-TAVR. Patients on ACEIs/ARBs had lower risk of all-cause mortality (RR: 0.74, 95% CI: 0.65-0.86, I^2^ = 62%, chi-square *P* < 0.01), cardiovascular mortality (RR: 0.70, 95% CI: 0.56-0.88, I^2^ = 0%, chi-square *P* = 0.54), and new-onset atrial fibrillation (RR: 0.71, 95% CI: 0.52-0.96, I^2^ = 0%, chi-square *P* = 0.59). Patients on ACEIs/ARBs had a similar risk of myocardial infarction, heart failure, stroke, new permanent pacemaker implantation, acute kidney injury, major bleeding, vascular complications, aortic regurgitation, and mitral regurgitation.

**Conclusions:**

We found that patients receiving ACEIs/ARBs had a lower risk of all-cause mortality, cardiovascular mortality, and new-onset atrial fibrillation. Risk of other outcomes was similar to patients not receiving ACEIs/ARBs. Randomized clinical trials are needed to explore the benefits of ACEIs/ARBs post-TAVR, so that definitive guidelines can be developed.

Transcatheter aortic valve replacement (TAVR) has become a definitive alternative to surgical aortic valve replacement for severe aortic stenosis (AS), across the spectrum of surgical risk.^1^

Severe AS is associated with left ventricular hypertrophy (LVH) that results in diastolic dysfunction. Excessive LVH, particularly when persisting after TAVR, is associated with worse prognosis.^2^^,^^3^ Renin-angiotensin-aldosterone system inhibitors, including angiotensin-converting enzyme inhibitors (ACEIs) and angiotensin receptor blockers (ARBs), are known to prevent adverse left ventricular modeling by reducing LVH and preventing myocardial fibrosis. Therefore, in theory, ACEIs/ARBs could be greatly beneficial for patients undergoing TAVR. This has been supported by large, registry-based observational studies demonstrating reduced mortality and heart failure (HF) readmissions in those receiving ACEIs/ARBs after aortic valve replacement.^3^, ^4^, ^5^ Yet, perhaps secondary to concerns regarding adverse effects, there remain no specific recommendations regarding the use of ACEIs/ARBs after TAVR. So, we sought to analyze existing evidence regarding the outcomes in patients receiving ACEIs/ARBs after TAVR.

## Methodology

We conducted this systematic review and meta-analysis according to the Preferred Reporting Items for Systematic Reviews and Meta-Analyses (PRISMA) guidelines.^6^ Our article is a meta-analysis of publicly available data, so institutional ethical approval was not required.

### Literature search and study selection

We performed a literature search of PubMed and Cochrane Library till June 14, 2023. We used key words ‘angiotensin-converting enzyme inhibitor’, ‘angiotensin receptor blocker’, ‘transcatheter aortic valve replacement’, and their word variations (Supplemental Methods).

We included all articles comparing outcomes of patients receiving ACEIs/ARBs after TAVR with outcomes of those not receiving ACEIs/ARBs. We did not impose any restrictions on language, sample size, or follow-up duration. We excluded editorials, review articles, and study designs.

After removing all duplicates, 2 investigators (V.B., A.K.) independently screened all articles based on titles and abstracts, and then conducted a full-text review. A third investigator (A.K.) resolved all conflicts.

### Data abstraction and quality assessment

From each included study, we abstracted data for the following variables: baseline characteristics of the participants, absolute events and sample sizes of outcomes of interest, and follow-up duration. All outcomes were abstracted on the longest available follow-up, and all data were abstracted on the intention-to-treat principle. We utilized the Newcastle-Ottawa Scale to assess biases associated with observational studies.^7^ This was carried out independently by V.B. and A.K., under the supervision of A.K.

### Outcome measures

Our primary outcome was all-cause mortality. Secondary outcomes were cardiovascular mortality, myocardial infarction (MI), HF, stroke/transient ischemic attack (TIA), new-onset atrial fibrillation (AF), new permanent pacemaker (PPM) requirement, acute kidney injury (AKI), major bleeding events, major vascular events, aortic regurgitation (AR), and mitral regurgitation (MR).

### Data synthesis and analysis

We calculated risk ratios (RR) and the corresponding 95% CI using the inverse variance method. We used the Paule-Mandel estimate for tau^2^ and Q-profile method for the CI of tau^2^ and tau. The Hartung-Knapp adjustment for random effects model was used. Heterogeneity was quantified with the I^2^ index, with 50% considered significant. Publication bias was assessed using visual inspection of the counter-enhanced funnel plot and statistically interpreted by Egger’s test. We considered *P* values of <0.05 as statistically significant. All analysis was carried out using R version 4.0.3.

## Results

### Characteristics of included studies

**Figure 1:**
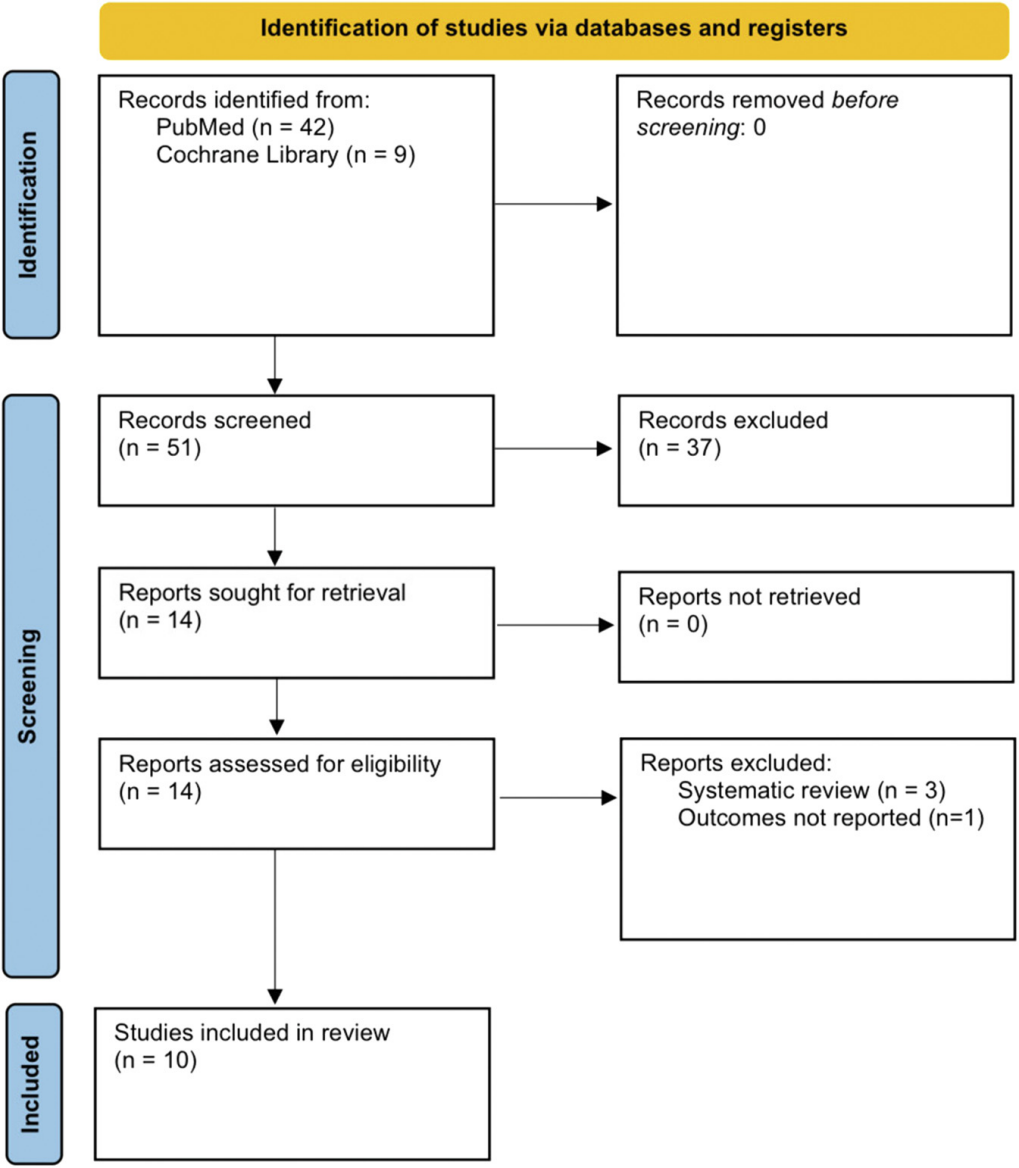
**PRISMA Flowchart for Study Search and Inclusion** PRISMA flowchart, showing search strategy, screening, and assessment for eligibility, leading to inclusion of 10 articles. PRISMA = Preferred Reporting Items for Systematic Reviews and Meta-Analyses.

Our initial search revealed 51 results. We finally included and analyzed results from 10 studies^3^^,^^4^^,^^8^, ^9^, ^10^, ^11^, ^12^, ^13^, ^14^, ^15^, ^16^, ^17^, ^18^, ^19^, ^20^, ^21^ (Figure 1, Table 1, Supplemental Table 2). Cumulatively, these studies compared 16,029 patients who received ACEIs/ARBs after TAVR, and 18,971 patients who did not.

**Table 1:** Definition of Outcomes in Included Studies

First Author, Year	All-Cause Mortality	Cardiovascular Mortality	HF	MI	Stroke/TIA	New Pacemaker Requirement
Inohara et al, 2018	Not specified (1 y)	Outcome not reported	Readmission for HF (1 y)	Not specified (during hospitalization)	Not specified (during hospitalization)	Outcome not reported
Kaewkes et al, 2020	VARC-2^16^ (2 y)	Outcome not reported	Hospitalization for HF (2 y)	VARC-2^16^ (2 y)	VARC-2^16^ (2 y)	Outcome not reported
Klinkhammer, 2019	VARC-2^16^ (2 y)	Outcome not reported	Change in symptoms consistent with HF necessitating additional pharmacotherapy/hospitalization (1 y)	VARC-2^16^ (1 y)	VARC-2^16^ (1 y)	VARC-2^16^ (periprocedural)
Ledwoch et al, 2020	VARC-2^16^ (3 y)	Outcome not reported	NYHA functional class III/IV (3 y)	VARC-2^16^ (3 y)	VARC-2^16^ (hospitalization)	VARC-2^16^ (hospitalization)
Ochiai et al, 2017	VARC-2^16^ (2 y)	Outcome not reported	Rehospitalization for HF (2 y)	VARC-2^16^ (periprocedural)	VARC-2^16^ (postprocedural)	VARC-2^16^ (postprocedural)
PARTNER 2, 2020	Not specified (2 y)	Not specified (2 y)	NYHA functional class III/IV (30 d)	Not specified (30 d)	Disabling stroke (30 d)	Not specified (30 d)
Phuong et al, 2021	Outcome not reported	Outcome not reported	Outcome not reported	Outcome not reported	Defined according to STS/ACC TVT registry^17^ (hospitalization)	Defined according to STS/ACC TVT registry^17^ (hospitalization)
Rodriguez-Gabella et al, 2019	VARC-2^16^ (3 y)	VARC-2^16^ (3 y)	NYHA functional class III/IV (3 y)	Outcome not reported	VARC-2^16^ (3 y)	VARC-2^16^ (hospitalization)s
Fischer-Rasokat et al, 2022	VARC-3^18^	VARC-3^18^	Outcome not reported	Outcome not reported	Outcome not reported	Outcome not reported
Cubeddu et al, 2023	Not specified (3 y)	Outcome not reported	Outcome not reported	Outcome not reported	Outcome not reported	Outcome not reported

First Author, Year	AF	AKI	Major Bleeding	Major Vascular Complication	AR	MR
Inohara et al, 2018	Outcome not reported	New dialysis requirement (hospitalization)	Outcome not reported	VARC-2;^16^ did not separate minor, major (hospitalization)	Moderate/severe AR (hospitalization)	Moderate/severe MR (hospitalization)
Kaewkes et al, 2020	New-onset (postprocedural)	Outcome not reported	Outcome not reported	Outcome not reported	Outcome not reported	Outcome not reported
Klinkhammer, 2019	Outcome not reported	Periprocedural increase in serum creatinine >1.5× baseline (periprocedural)	VARC-2^16^ (periprocedural)	VARC-2^16^ (periprocedural)	VARC-2^16^, moderate or severe AR (1 y)	Moderate/severe MR (1 y)
Ledwoch et al, 2020	Outcome not reported	VARC-2^16^, AKI stage 1, 2, 3 (hospitalization)	VARC-2^16^ (hospitalization)	VARC-2^1^ (hospitalization)	AR grade ≥II, according to society guidelines^19^, ^20^, ^21^ (hospitalization)	Outcome not reported
Ochiai et al, 2017	New-onset (postprocedural)	VARC-2^16^ – AKI stage 1, 2, 3 (postprocedural)	VARC-2^16^, major or life threatening bleeding (postprocedural)	VARC-2^16^ (postprocedural)	VARC-2^16^,≥ moderate AR (postprocedural)	≥Moderate MR (postprocedural)
PARTNER 2, 2020	Outcome not reported	VARC-2^16^ (30 d)	VARC-2^16^, ‘life threatening or disabling bleeding’ (30 d)	VARC-2^16^ (30 d)	Moderate or severe paravalvular AR (30 d)	Moderate or severe MR (30 d)
Phuong et al, 2021	Outcome not reported	Increase in creatinine ≥0.30 mg/dL at any time between TAVR and discharge (hospitalization)	Bleeding that leads to ≥3 g/dL loss; whole blood or packed red blood cells; procedure/intervention/surgery to stop bleeding (hospitalization)	Outcome not reported	Outcome not reported	Outcome not reported
Rodriguez-Gabella et al, 2019	New-onset (at discharge)	Outcome not reported	VARC-2^16^ (hospitalization)	VARC-2^16^ (hospitalization)	VARC-2^16^, moderate or severe AR (hospitalization)	Outcome not reported
Fischer-Rasokat et al, 2022	Outcome not reported	Outcome not reported	Outcome not reported	Outcome not reported	Moderate AR (postprocedural)	Outcome not reported
Cubeddu et al, 2023	Outcome not reported	Outcome not reported	Outcome not reported	Outcome not reported	Outcome not reported	Outcome not reported

AKI = acute kidney injury; AR = aortic regurgitation; HF = heart failure; MI = myocardial infarction; MR = mitral regurgitation; STS/ACC TVT = Society of Thoracic Surgeons/American College of Cardiology Transcatheter Valve Therapy; TIA = transient ischemic attack; VARC = Vascular Academic Research Consortium.

### Baseline characteristics

All included studies involved patients with a mean age of around 80 years, and most had a male predominance. The vast majority of patients in each study were hypertensive. The proportion of patients with HF ranged from around 50% to over 90%, while the proportion of those with chronic kidney disease ranged from around 35% to around 75%. Among those studies that reported it, the mean Society of Thoracic Surgeons Predicted Risk of Mortality score ranged from 4.9% (intermediate risk) to 7.7% (high risk). Only 3 studies reported the proportion of patients who were on ACEIs/ARBs prior to TAVR, with varying percentages (Table 2).

**Table 2:** Baseline Characteristics of Patients in Included Studies

	Ochiai et al, 2017		Inohara et al, 2018		Rodriguez-Gabella et al, 2019		Klinkhammer, 2019		Cubeddu et al, 2023	
Design	R, PSM		R, PSM		R, PSM		R		R	
Country	Japan		USA		Spain		USA		USA	
Characteristic	I	C	I	C	I	C	I	C	I	C
N	371	189	7,948^a^	7,948^a^	695^a^	695^a^	71	98	3,172	5,840
Age (y)	84.2	84.8	82.4	82.4	80.8	80.6	77.8	80.1	79.3	80.4
Male	29.9	24.3	52.3	51.6	46.3	46.2	56	61	51.9	54.4
BMI (kg/m^2^)	22.6	21.5	28.3	28.3	27.9	27.5	31.0	29.9	-	-
STS PROM	7.0	7.0	7.5	7.5	5.3	5.3	-	-	-	-
HTN	83.8	61.4	93.6	93.1	78.4	78.0	96	77	97.5	92.8
CAD	41.5	33.9	-	-	40.6	41.2	75	77	86.8	86.4
HF	46.6	54.5	79.9	79.5	66.8	67.9	42	50	49.6	48.9
DM	27.8	24.3	38.7	38.7	33.4	35.4	51	32	50.6	41.8
CKD	66.3	57.1	46.7	46.2	41.9	42.9	46	56	35.7	36.9
Past stroke/TIA	12.4	14.3	21.3	21.7	11.4	13.5	10	13	-	-
DLP	-	-	-	-	54.8	53.5	94	85	-	-
Past MI	9.4	5.3	24.2	24.0	15.8	15.5	-	-	17.2	17.0
PVD	15.1	9.5	-	-	11.4	15.4	18	23	-	-
COPD	19.4	14.3	24.9	24.6	23.2	24.6	-	-	-	-
Pacemaker	-	-	17.8	18.2	8.8	8.2	8	14	-	-
Past PCI	31.5	23.3	-	-	22.9	25.2	32	42	-	-
Past CABG	7.5	5.8	28.8	28.5	8.4	9.2	34	34	-	-
AV procedure	-	-	12.5	12.5	5.5	6.0	17	24	-	-
ACEI	-	-	66	0	-	-	42	15	-	-
ARB	-	-	35.5	0	-	-	29	2	-	-
Statin	49.9	37.6	-	-	-	-	77	67	-	-
Aspirin	84.9	90.5	-	-	-	-	80	78	-	-
Beta-blocker	33.7	27.5	-	-	-	-	83	81	-	-

	PARTNER 2, 2020		Ledwoch et al, 2020		Kaewkes et al, 2020		Phuong et al, 2021		Fischer-Rasokat et al, 2022	
Design	R, PSM		R		R		R		R, PSM	
Country	USA, Canada		Germany		USA		USA		Germany	
Characteristic	I	C	I	C	I	C	I	C	I	C
N	1,736	2,243	225	98	877	807	308	427	626^a^	626^a^
Age (y)	81.7	82.9	80.7	79.8	82.3	80.6	77.9	79.3	82.1	81.9
Male	60.8	58.4	56	54	60	57.6	54.9	54.1	44.2	47.1
BMI (kg/m^2^)	28.8	27.8	25.7	25	26.6	27.7	30.5	29.1	26.5	26.6
STS PROM	7.2	7.7	-	-	5.1	4.9	5.7	6.9		
HTN	96.3	89.7	94.2	81	88.9	93.7	93.5	85.7	85.8	82.7
CAD	81.0	76.0	72	60	45.2	51.1	-	-	57.8	53.5
HF	82.7	84.1	26.2	70	92.9	94.2	-	-	79.1	79.1
DM	40.6	32.5	25.8	19	28.6	37.5	44.5	36.5	32.6	32.7
CKD	-	-	53.6	63	77.5	79.2	-	-		
Past stroke/TIA	18.7	18.9	12.9	13	17.4	16.7	14.9	15.7	15.2	12.1
DLP	84.4	79.4	65.6	52	-	-	-	-		
Past MI	21.0	17.1	20.9	7	10.9	12.1	24.7	23.2	11.8	10.1
PVD	34.7	32.4	19.1	19	21.8	23.0	27.3	24.4	10.7	10.7
COPD	34.7	31.8	13.0	16	22.7	17.6	16.9	30.0	17.4	19.3
Pacemaker	-	-	14.7	14	-	-	14.6	8.9	-	-
Past PCI	33.8	30.8	53.4	37	21.8	23.0	-	-	-	-
Past CABG	-	-	4.9	10	17.6	21.7	-	-	-	-
AV procedure	-	-	-	-	-	-	-	-	-	-
ACEI	-	-	-	-	-	-	90.6	25.5	-	-
ARB	-	-	-	-	-	-			-	-
Statin	-	-	-	-	-	-	-	-	-	-
Aspirin	-	-	-	-	-	-	-	-	-	-
Beta-blocker	-	-	-	-	-	-	-	-	-	-

ACEI = angiotensin-converting enzyme inhibitor; ARB = angiotensin receptor blocker; AV = aortic valve; BMI = body mass index; C = control (not receiving ACEIs/ARBs); CABG = coronary artery bypass graft; CAD = coronary artery disease; CKD = chronic kidney disease; COPD = chronic obstructive pulmonary disease; DLP = dyslipidemia; DM = diabetes mellitus; HF = heart failure; HTN = hypertension; I = intervention (receiving ACEIs/ARBs); MI = myocardial infarction; PCI = percutaneous intervention; PSM = propensity score matched; PVD = peripheral vascular disease; R = retrospective cohort; STS PROM = Society of Thoracic Surgeons Predicted Risk of Mortality; TIA = transient ischemic attack.

a Matched population.

### Primary outcome

**Figure 2:**
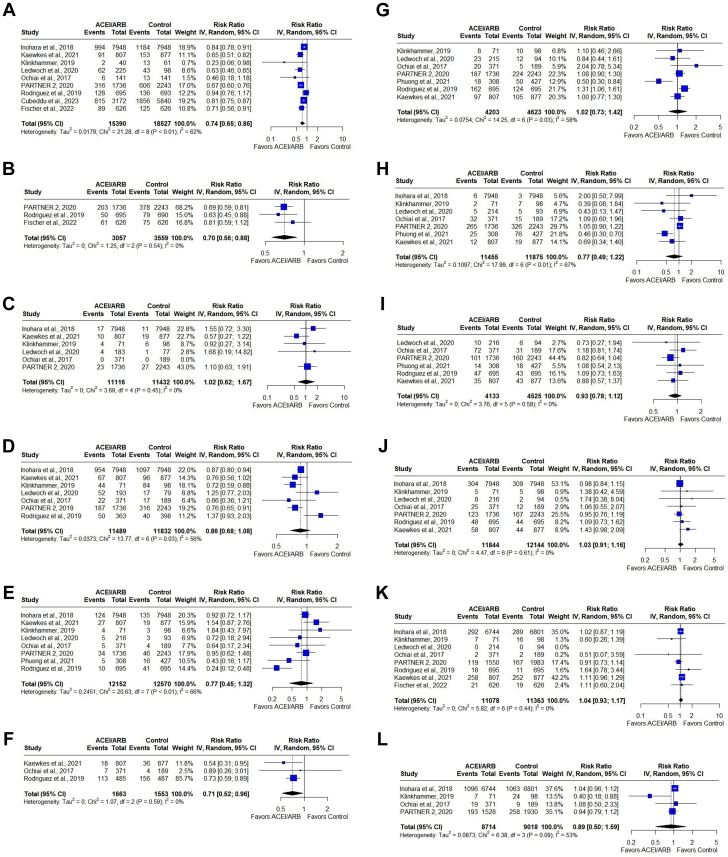
Meta-Analysis of Outcomes Forest plot comparing outcomes between patients receiving ACEIs/ARBs (intervention) with those not receiving ACEIs/ARBs after TAVR (control). Outcomes: (A) all-cause mortality, (B) cardiovascular mortality, (C) myocardial infarction, (D) heart failure, (E) stroke/TIA, (F) new-onset atrial fibrillation, (G) new permanent pacemaker requirement, (H) acute kidney injury, (I) major bleeding, (J) major vascular complications, (K) aortic regurgitation, (L) mitral regurgitation. ACEI = angiotensin-converting enzyme inhibitor; ARB = angiotensin receptor blocker; TAVR = transcatheter aortic valve replacement; TIA = transient ischemic attack.

**Figure 3:**
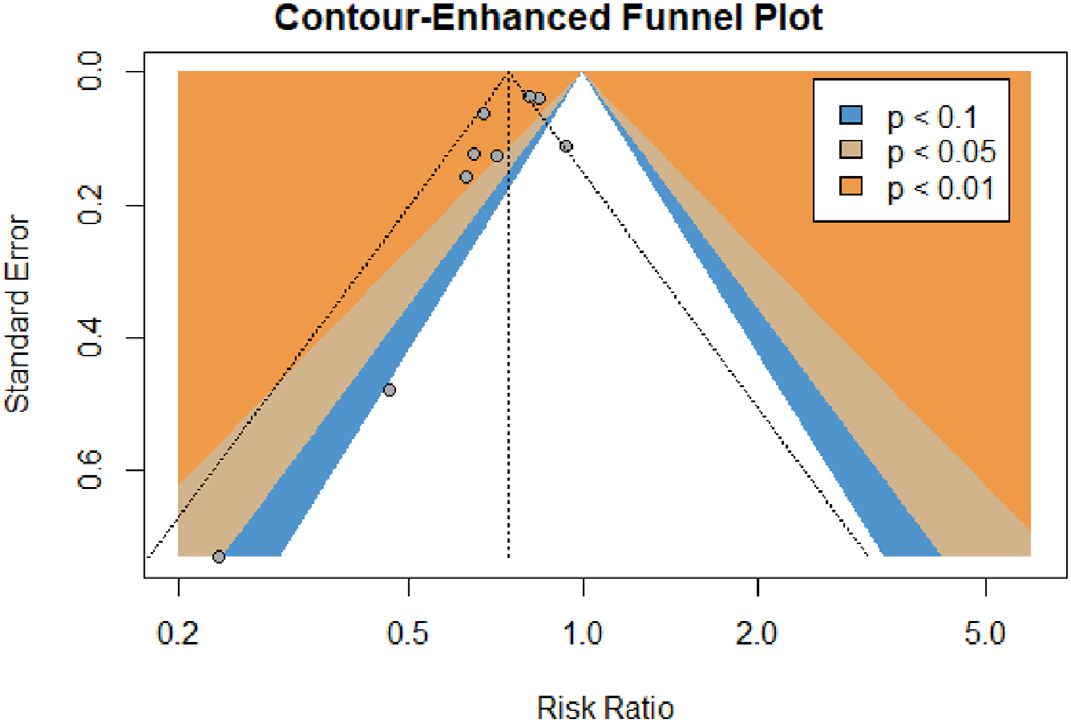
**Counter-Enhanced Funnel Plot for Primary Outcome: All-Cause Mortality** Counter-enhanced funnel plot showing asymmetry with missing studies, with high standard error and risk ratio higher than the pooled estimate.

Nine of ten studies reported all-cause mortality. Patients treated with ACEIs/ARBs had a lower risk of all-cause mortality compared with patients not receiving ACEIs/ARBs (RR: 0.74, 95% CI: 0.65-0.86, I^2^ = 62%, chi-square *P* < 0.01) (Figure 2A). Visual inspection of the counter-enhanced funnel plot revealed asymmetry with missing studies, with high standard error, and RR higher than the pooled estimate (Figure 3). Egger’s test, however, reported an intercept of −1.56 (95% CI: −3.01 to 0.10), t-value = −2.09 and *P* = 0.07, indicating no significant publication bias.

### Secondary outcomes

Cardiovascular mortality: Only 3 studies reported cardiovascular mortality. Patients treated with ACEIs/ARBs had a lower risk of cardiovascular mortality compared with patients not receiving ACEIs/ARBs (RR: 0.70, 95% CI: 0.56-0.88, I^2^ = 0%, chi-square *P* = 0.54) (Figure 2B).

MI: 6 studies reported MI. Risk was similar in both groups (RR: 1.02, 95% CI: 0.62-1.67, I^2^ = 0%, chi-square *P* = 0.45) (Figure 2C).

HF: 7 studies reported HF. Patients treated with ACEIs/ARBs had a similar risk of HF compared with patients not receiving ACEIs/ARBs (RR: 0.86, 95% CI: 0.68-1.08, I^2^ = 56%, chi-square *P* = 0.03) (Figure 2D).

Stroke/TIA: 8 studies reported stroke/TIA. Risk of stroke/TIA was similar in both groups (RR: 0.77, 95% CI: 0.45-1.32, I^2^ = 66%, chi-square *P* = < 0.01) (Figure 2E).

New-onset AF: 3 studies reported new-onset AF. Risk was lower in patients treated with ACEIs/ARBs compared with patients not receiving ACEIs/ARBs (RR: 0.71, 95% CI: 0.52-0.96, I^2^ = 0%, chi-square *P* = 0.59) (Figure 2F).

New PPM requirement: 7 studies reported new PPM requirement. Risk of pacemaker requirement was similar in both groups (RR: 1.02, 95% CI: 0.73-1.42, I^2^ = 58%, chi-square *P* = 0.03) (Figure 2G).

AKI: 7 studies reported AKI. Risk of AKI was similar between the 2 groups (RR: 0.77, 95% CI: 0.49-1.22, I^2^ = 67%, chi-square *P* < 0.01) (Figure 2H).

Major bleeding: 6 studies reported major bleeding. Risk of major bleeding was similar in both groups (RR: 0.93, 95% CI: 0.78-1.12, I^2^ = 0%, chi-square *P* = 0.58) (Figure 2I).

Major vascular complications: 7 studies reported major vascular complications. Risk was similar in both groups (RR: 1.03, 95% CI: 0.91-1.16, I^2^ = 0%, chi-square *P* = 0.61) (Figure 2J).

AR: 8 studies reported AR. Risk of AR was similar in both groups (RR: 1.04, 95% CI: 0.93-1.17, I^2^ = 0%, chi-square *P* = 0.44) (Figure 2K).

MR: 4 studies reported MR. Risk was similar in both groups (RR: 0.89, 95% CI: 0.50-1.59, I^2^ = 53%, chi-square *P* = 0.09) (Figure 2L).

### Risk of bias

According to Newcastle-Ottawa Scale for cohort studies, scores for the included studies ranged from 6 to 9. Most of these studies scored 9. For the other studies, ensuring adequate comparability was unsatisfactory, with 2 other studies additionally having unsatisfactory length and adequacy of follow-up, respectively (Supplemental Table 1).

## Discussion

In our meta-analysis comparing clinical outcomes of patients with and without ACEIs/ARBs after TAVR, we found that patients receiving ACEIs/ARBs had a lower risk of both all-cause and cardiovascular mortality, as well as postprocedural, new-onset AF (Central Illustration). There were no differences in risk of other secondary outcomes, including AKI. These findings of clinical benefit without greater risk of adverse events provide further evidence supporting the use of ACEIs/ARBs after TAVR, a practice which still lacks consensus.

**Central Illustration:**
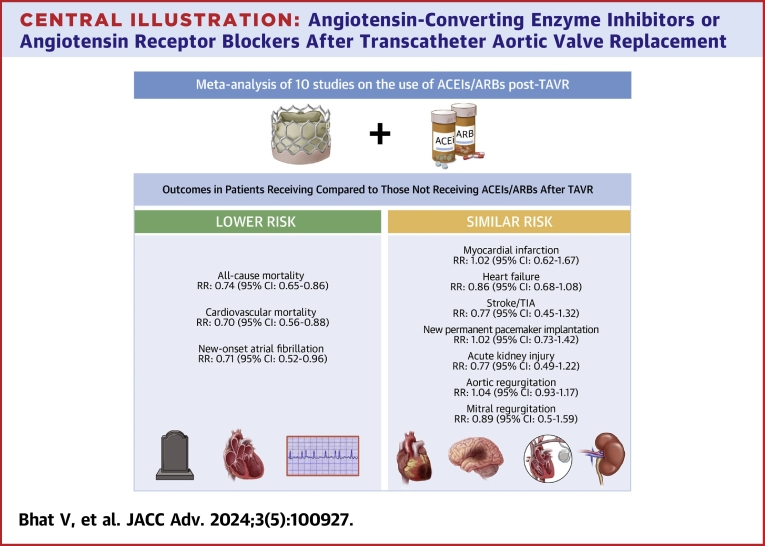
**Angiotensin-Converting Enzyme Inhibitors or Angiotensin Receptor Blockers After Transcatheter Aortic Valve Replacement** Meta-analysis of 10 studies comparing outcomes of patients receiving ACEIs/ARBs after TAVR (intervention), compared with those who did not (control). The intervention group had lower all-cause mortality, cardiovascular mortality, and new-onset atrial fibrillation compared with the control group. ACEI = angiotensin-converting enzyme inhibitor; ARB = angiotensin receptor blocker; TAVR = transcatheter aortic valve replacement; TIA = transient ischemic attack.

AS involves the entire heart, not just the aortic valve. Central to its pathology is compensatory LVH, which results in compromised subendocardial perfusion, causing myocardial fibrosis.^5^^,^^22^ Progression of this fibrosis, along with myocyte necrosis eventually cause decompensation and HF.^22^^,^^23^ Given that angiotensin II and aldosterone are key mediators of these pathological processes, ACEIs/ARBs could aid outcomes.^24^^,^^25^ Yet, for a long time, ACEIs/ARBs were avoided in AS for fear of severe hypotension in the setting of ventricular outflow obstruction.^3^^,^^26^ With time, clinical evidence of good tolerability, as well as delayed progression of AS and regression of LVH accumulated, proving earlier fears were unfounded. ACEIs/ARBs are now considered for AS before replacement,^24^ but there are no guidelines regarding their use after TAVR.

Aortic valve replacement has been shown to rapidly result in regression of cellular hypertrophy and diffuse interstitial fibrosis. However, focal replacement fibrosis, which correlates with risk of decompensation, remains permanent. Patients who undergo TAVR continue to have lower survival compared with the general population.^27^ This highlights the need to identify optimal medical therapy after TAVR to improve these patients’ outcomes. ACEIs/ARBs could accelerate the regression of LVH and reversal of adverse remodeling, a hypothesis that was supported by the analysis of the OCEAN-TAVI (Optimized transCathEter vAlvular iNtervention) registry, where patients receiving ACEIs/ARBs after TAVR had greater LV mass regression compared with those who did not.^8^ Rapid regression of LVH has been associated with decreased readmissions, particularly due to HF, after TAVR.^28^ Additional benefits of ACEIs/ARBs after TAVR have been postulated—these include sympathetic modulation, further reducing the risk of HF, and an antiarrhythmic benefit due to their potassium channel-sparing action.^24^

In line with these expected benefits, we found reduced mortality, both cardiovascular and all-cause, in patients receiving ACEIs/ARBs. This is in line with the included observational studies and prior meta-analyses,^3^^,^^4^^,^^29^, ^30^, ^31^, ^32^ further strengthening available evidence regarding the use of ACEIs/ARBs after TAVR. Additionally, we found a reduction in postprocedural AF. AF after TAVR increases mortality;^30^^,^^33^ its reduced incidence could have contributed to decreased mortality in those receiving ACEIs/ARBs.

However, we did not find a significant reduction in HF among those receiving ACEIs/ARBs. HF is the principal cause for repeat hospitalizations after TAVR, particularly within the first year,^8^^,^^28^ with almost 25% of patients undergoing TAVR undergoing HF-related readmissions at some point.^34^ Our findings contrast with the Inohara et al^4^ registry analysis and the Sun et al^30^ similar meta-analysis. We also did not find any significant difference in other secondary outcomes, such as PPM implantation, or paravalvular AR, both of which contribute to increased mortality after TAVR. This is probably since arrhythmias requiring PPM after TAVR commonly result from structural damage to the conduction system,^35^ and AR after TAVR commonly results from valve sizing or placement issues.^36^ Finally, the lack of a significant difference in the risk of AKI between the 2 groups should alleviate concerns regarding the safety of these drugs after TAVR.

### Study limitations

Our study is not without limitations. This was a study-level meta-analysis, so was limited in its ability to identify heterogeneity between studies. Our findings are based on data from observational studies, with no randomized controlled trials, potentially allowing more bias. Our analysis does not account for the premorbid state of patients, or whether they were on ACEIs/ARBs prior to TAVR. Furthermore, the length of follow-up varied from study to study. While we found a reduced risk of cardiovascular mortality and AF, this was based on only 3 studies that reported these outcomes, so cautious interpretation may be warranted. We did not analyze outcomes based on patients’ baseline left ventricular ejection fraction (LVEF). While Chen et al^3^ found mortality benefit regardless of LVEF, Inohara et al^4^ found benefit only in those with preserved LVEF, while Fischer-Rasokat et al^15^ found greater benefit in those with LVEF <40%. Therefore, future analyses utilizing a uniform definition of ‘reduced’ and ‘preserved’ LVEF are warranted. We also included all patients receiving ACEIs/ARBs, regardless of the dose received. Finally, the clinical definitions used by each study (eg, for stroke, AKI) varied slightly (Table 1). However, we have used the most recent available data and used the random effects model to account for heterogeneity, which was lacking in prior analyses.^29^^,^^31^

## Conclusions

We found that patients who received ACEIs/ARBs after TAVR had a lower risk of all-cause mortality, cardiovascular mortality, and new-onset AF, compared with those who did not. Robust clinical trial data, such as that from the ongoing RASTAVI (RAS blockade after TAVI; NCT03201185) and ARISTOTE (Angiotensin Receptor Blocker Following aortIc Valve Intervention for Aortic STenOsis: a Randomized mulTi-cEntric Double-blind Phase II Study; NCT03315832) trials will provide more conclusive evidence for stakeholders (Supplemental Table 3).

PERSPECTIVES **COMPETENCY IN MEDICAL KNOWLEDGE:** Our study provides further evidence regarding the benefits of using ACEIs/ARBs after TAVR.

**COMPETENCY IN PATIENT CARE:** Our meta-analysis of over 30,000 patients undergoing TAVR demonstrated that those receiving ACEIs had both reduced mortality and AF, with no increased risk of adverse events.

**TRANSLATIONAL OUTLOOK:** Randomized clinical trials are required (are ongoing) to provide definitive evidence and inform clinical guidelines regarding the use of ACEIs/ARBs after TAVR.

## Funding support and author disclosures

This work was funded by makeadent.org Ram and Sanjita Kalra Aavishqaar Fund. There is no role of the funder in design and conduct of the study, collection, analysis, and interpretation of the data, or in preparation, review, or approval of the manuscript. Dr Kalra is the founder and director of makeadent.org, a 501(c)3 registered non-profit organization. All other authors have reported that they have no relationships relevant to the contents of this paper to disclose.
