# Supplementary material for: Angiotensin-Converting Enzyme Inhibitors or Angiotensin Receptor Blockers After Transcatheter Aortic Valve Replacement: A Meta-Analysis
Source: JACC Adv. 2024 Apr 6;3(5):100927. doi: 10.1016/j.jacadv.2024.100927 (PMC11198320; doi:10.1016/j.jacadv.2024.100927)
Supplement: Supplementary data [file mmc1.docx]

**SUPPLEMENTAL APPENDIX**

**Supplemental Methods: Search strategy**

**PubMed:** (((renin-angiotensin inhibitor) OR (angiotensin receptor blocker) OR (angiotensin converting enzyme inhibitor)) AND ((transcatheter aortic valve replacement) OR (transcatheter aortic valve implantation))) [Title/Abstract]

**Cochrane Library:** (((renin-angiotensin inhibitor) OR (angiotensin receptor blocker) OR (angiotensin converting enzyme inhibitor)) AND ((transcatheter aortic valve replacement) OR (transcatheter aortic valve implantation))) in Title Abstract Keyword

Search last carried out on June 14, 2023

**Supplemental Table 1: Risk of bias (Newcastle-Ottawa Quality Assessment Scale)**

| **Study name** | **Exposed cohort representativeness** | **Non-exposed cohort** | **Exposure ascertainment** | **Outcome^a^** | **Cohort comparability** | **Outcome assessment** | **Follow-up length^b^** | **Follow-up adequacy** | **Score** |
| --- | --- | --- | --- | --- | --- | --- | --- | --- | --- |
| Inohara et al., 2018 | 1 | 1 | 1 | 1 | 2 | 1 | 1 | 1 | 9 |
| Kaewkes et al., 2020 | 1 | 1 | 1 | 1 | 0 | 1 | 1 | 1 | 7 |
| Klinkhammer, 2019 | 1 | 1 | 1 | 1 | 0 | 1 | 1 | 0^c^ | 6 |
| Ledwoch et al., 2020 | 1 | 1 | 1 | 1 | 0 | 1 | 1 | 1 | 7 |
| Ochiai et al., 2017 | 1 | 1 | 1 | 1 | 2 | 1 | 1 | 1 | 9 |
| PARTNER 2, 2020 | 1 | 1 | 1 | 1 | 2 | 1 | 1 | 1 | 9 |
| Phuong et al., 2021 | 1 | 1 | 1 | 1 | 0 | 1 | 0^d^ | 1 | 6 |
| Rodriguez-Gabella et al., 2019 | 1 | 1 | 1 | 1 | 2 | 1 | 1 | 1 | 9 |
| Fischer-Rasokat et al., 2022 | 1 | 1 | 1 | 1 | 2 | 1 | 1 | 1 | 9 |
| Cubbedu et al., 2023 | 1 | 1 | 1 | 1 | 2 | 1 | 1 | 1 | 9 |

1. Outcome – all-cause mortality, except Phuong et al., who did not report it
2. Adequate length – 1 year or more
3. Survival at two years – available only for 40/71 (exposed), 61/98 (non-exposed)
4. Outcomes reported only till discharge

**Supplemental Table 2: Inclusion and exclusion criteria of included studies**

| Study | Inclusion criteria | Exclusion criteria |
| --- | --- | --- |
| Inohara et al., 2018 | - >65 years old - With Medicare - TAVR between July 2014 and January 2016 - Derived from STS/ ACC TVT registry | - Died during index hospitalization - Discharged against medical advice, transferred to another acute care hospital, or transferred to hospice care - Had contraindication to the use of both ACEIs and ARBs or had missing data for prescription of both ACEIs and ARBs - <65 years old - Could not be linked to Centers for Medicare & Medicaid Services administrative claims |
| Kaewkes et al., 2020 | - Native severe AS - TAVR from January 2013 to November 2017 - At Cedars-Sinai Medical Center, USA | - Died during index hospitalization - Discharged against medical advice - Were referred to other hospitals or hospice care - Had a contraindication for beta blockers or ACEIs/ARBs - Data of medication at discharge was missing |
| Klinkhammer, 2019 | - Severe symptomatic AS - TAVR from August 2012 to November 2016 - At Sanford Health, USA. | - Less than 1 year of post-TAVR follow-up |
| Ledwoch et al., 2020 | - Successful TAVR between January 2015 and September 2019 - Informed written consent | - None |
| Ochiai et al., 2017 | - Severe symptomatic AS undergoing TAVR between October 2013 and April 2016 - Derived from OCEAN-TAVI registry (Japan) | - Death within 6 months of TAVR - Lack of data from 6-month follow-up visit - Only one prescription of ACEIs/ARBs during follow-up |
| PARTNER 2, 2020 | - Cohort undergoing TAVR in PARTNER 2 studies [1,2] – patients with severe AS, at intermediate, high or prohibitively high risk of surgery | - Baseline creatinine >3 mg/dL or renal replacement therapy - Congenital bicuspid aortic valve - Severe AR - LVEF <20% - Estimated life expectancy of <2 years |
| Phuong et al., 2021 | - Severe symptomatic AS - TAVR between January 2014 and March 2019 - At University of Vermont Medical Center, USA | Not specified |
| Rodriguez-Gabella et al., 2019 | - TAVR between August 2007 and August 2017 - Derived from RASTAVI registry (Spain) | Not specified |
| Cubbedu et al., 2023 | - TAVR from January 2014 to December 2018. - Must be enrolled in commercial or Medicare Advantage plan for >6 months prior to admission. - Derived from Optum® Clinformatics® Data Mar (USA). | - Death <90 days from TAVR. - Transapical access TAVR. - ESRD - Missing region data. |
| Fischer-Rasokat et al., 2022 | - Transfemoral TAVR for severe AS (as per 2021 ESC/ EACTS guidelines) [3] - At Kerckhoff Heart Center, Benekestr, Germany, from January 2011 to December 2020 | - Intraprocedural conversion to open-heart surgery. - In-hospital death |

ACEIs – angiotensin converting enzyme inhibitors; ARBs – angiotensin receptor blockers; AR – aortic regurgitation; AS – aortic stenosis; STS/ ACC TVT - Society of Thoracic Surgeons/ American College of Cardiology Transcatheter Valve Therapy; ESC/ EACTS – European Society of Cardiology/ European Association for Cardio-Thoracic Surgery; ESRD – end-stage renal disease; LVEF – left ventricular ejection fraction; TAVR – transcatheter aortic valve replacement;

**References:**

1. Leon MB, Smith CR, Mack MJ, et al. Transcatheter or Surgical Aortic-Valve Replacement in Intermediate-Risk Patients. N Engl J Med. 2016;374(17):1609-1620. doi:10.1056/NEJMoa1514616
2. Kodali S, Thourani VH, White J, et al. Early clinical and echocardiographic outcomes after SAPIEN 3 transcatheter aortic valve replacement in inoperable, high-risk and intermediate-risk patients with aortic stenosis. Eur Heart J. 2016;37(28):2252-2262. doi:10.1093/eurheartj/ehw112
3. Vahanian A, Beyersdorf F, Praz F, et al. 2021 ESC/EACTS Guidelines for the management of valvular heart disease [published correction appears in Eur Heart J. 2022 Feb 18;:]. Eur Heart J. 2022;43(7):561-632. doi:10.1093/eurheartj/ehab395

**Supplemental Table 3: Ongoing randomized control trials investigating ACEIs/ ARBs after TAVR**

|  | **RASTAVI -** Renin-angiotensin System Blockade Benefits in Clinical Evolution and Ventricular Remodeling After Transcatheter Aortic Valve Implantation [1] | **ARISTOTE -** Efficacy of Angiotensin Receptor Blocker Following aortIc Valve Intervention for Aortic STenOsis: a Randomized mulTi-cEntric Double-blind Phase II Study [2] |
| --- | --- | --- |
| Identifier | NCT03201185 | NCT03315832 |
| Country | Spain | France |
| Phase | IV | II |
| Inclusion criteria | Adults undergoing TAVR for severe AS | - Men ≥18 years old. - Postmenopausal women - absence of menses for the last 12 months without alternative medical cause. - Severe AS (aortic valve area <1.0cm² or <0.6cm²/m² and aortic mean pressure gradient ≥40mmHg or aortic maximal velocity >4m/s, as assessed using transthoracic echocardiography). - Indication for aortic valve intervention (TAVR/SAVR). - Affiliation to the French Social Security system. |
| Exclusion criteria | - Severe mitral valvulopathy - LVEF <40% with myocardial infarction or dilated cardiomyopathy - Patients on an ACEI/ ARB in the last 3 months - History of hypersensitivity or allergy to any of the study drugs, or drugs of similar chemical classes; known or suspected contraindications to the study drugs - Non-MR-conditional cardiac devices - Estimated GFR <30 ml/min - Systolic blood pressure <100 mmHg or diastolic <40 mmHg - Pregnant women - Participating in other investigational trial at the time of enrolment | - Patients already on ACEI/ ARBs prior to randomization. - Concomitant CABG or other valvular intervention - Other significant left-sided valvular heart diseases (≥moderate), even without concomitant procedure - Any contraindication to CMR - Chronic kidney disease with estimated GFR <30 ml/min - Prior or planned organ transplantation - Hyperkalemia (>5.5 mmol/L) at inclusion visit. - Severe hepatic failure, biliary cirrhosis, cholestasis - Combined use of aliskiren - Concomitant diabetes mellitus or renal failure with GFR <60mL/min/1.73m² - Systolic blood pressure <100 mmHg - History of angioedema - History of hypersensitivity or allergy to ARBs - Under legal authority - Patients not under ACEIs/ ARBs prior to randomization but who should benefit from this treatment to improve outcome:  1. Heart failure with reduced ejection fraction (<40%) 2. Coronary artery disease 3. Clinical peripheral artery disease 4. History of cerebrovascular disease 5. Uncontrolled hypertension despite use of other therapeutic classes 6. Diabetes mellitus  - Impossibility to perform randomization into the 9-day post-intervention period due to per procedural complication with prolonged stay in intensive cardiac care unit (including death) |
| Intervention | Ramipril – 2.5mg daily, up-titrated till 10mg daily (or maximum tolerated dose) | Valsartan – 80mg daily, up-titrated till 160mg daily |
| Comparator | Placebo | Placebo |
| Primary outcome | First occurrence of cardiovascular events - cardiac death or heart failure hospitalization or stroke. | Indexed LV mass at 12 months, assessed by CMR |
| Secondary outcomes | - Changes in left ventricular remodeling at 12 months assessed by cardiac MRI. - Myocardial fibrosis will be measured in grams - Change in functional capacity from baseline at 12 months, assessed by six minute walk test. - Death due to cardiac causes, at 12 and 36 months. - Admissions due to heart failure, at 12 and 36 months. - Stroke, at 12 and 36 months - All-cause mortality, at 12 and 36 months | - LV global longitudinal strain, at 12 months, using CMR and TTE - Left atrial volume at 12 months, using CMR and TTE - Indexed LV mass at 12 months, using TTE - Native T1 change at 12 months using CMR - Rate of LGE at 12 months on CMR - Volume of LGE change at 12 months on CMR - Extra cellular volume change at 12 months on CMR - Indexed interstitial volume change at 12 months on CMR - Change in electrocardiographic strain at 12 months - Change in LVEF at 12 months using CMR and TTE - Peak exercise VO2 at 12 months. - VE/VCO2 ratio at 12 months. - Maximal load reached at 12 months - NYHA functional class at 12 months - Exercise oscillatory ventilation rate at 12 months. - Nt-proBNP change at 12 months - Change in plasma cardiac troponin I at 12 months - Incidence of adverse events till 13 months |

ACEIs – angiotensin converting enzyme inhibitors; ARBs – angiotensin receptor blockers; AS – aortic stenosis; CABG – coronary artery bypass graft; CMR – cardiac magnetic resonance imaging; GFR – glomerular filtration rate; LGE - Late gadolinium enhancement; LV – left ventricle; LVEF – left ventricular ejection fraction; NYHA – New York Heart Association; SAVR – surgical aortic valve replacement; TAVR – transcatheter aortic valve replacement; TTE – transthoracic echocardiography

**References:**

1. Renin-angiotensin System Blockade Benefits in Clinical Evolution and Ventricular Remodeling After Transcatheter Aortic Valve Implantation (RASTAVI) (RASTAVI) ClinicalTrials.gov identifier: NCT03201185. Updated September 24, 2021. Accessed June 10, 2023. <https://clinicaltrials.gov/ct2/show/NCT03201185>
2. Efficacy of Angiotensin Receptor Blocker Following aortIc Valve Intervention for Aortic STenOsis: a Randomized mulTi-cEntric Double-blind Phase II Study (ARISTOTE). ClinicalTrials.gov identifier: NCT03201185. Updated March 17, 2023. Accessed October 7, 2023. <https://clinicaltrials.gov/study/NCT03315832>
